# Hedonicity in functional motor disorders: a chemosensory study assessing taste

**DOI:** 10.1007/s00702-020-02244-5

**Published:** 2020-08-28

**Authors:** Maria Paola Cecchini, Stefano Tamburin, Alice Zanini, Federico Boschi, Benedetta Demartini, Diana Goeta, Carlo Dallocchio, Angela Marotta, Mirta Fiorio, Michele Tinazzi

**Affiliations:** 1 Department of Neurosciences, Biomedicine and Movement Sciences, Anatomy and Histology Section, University of Verona, Strada le Grazie 8, 37134 Verona, Italy; 2 Department of Neurosciences, Biomedicine and Movement Sciences, Neurology Section, Verona University Hospital, University of Verona, Piazzale Scuro 10, 37134 Verona, Italy; 3 Department of Computer Science, University of Verona, Verona, Italy; 4 Psychiatry Unit II, A.O. San Paolo, ASST Santi Paolo e Carlo, Milan, Italy; 5 Neurology Unit, Department of Medical Area, ASST Pavia, Pavia, Italy; 6 Department of Neurosciences, Biomedicine and Movement Sciences, University of Verona, Verona, Italy

**Keywords:** Taste, Hedonicity, Functional motor disorders (FMD)

## Abstract

The aim of this study was to explore hedonicity to basic tastes in patients with functional motor disorders (FMDs) that are often associated with impairment in emotional processing. We recruited 20 FMD patients and 24 healthy subjects, matched for age and sex. Subjects were asked to rate the hedonic sensation (i.e., pleasant, neutral, and unpleasant) on a − 10 to +10 scale to the four basic tastes (sweet, sour, salty, and bitter) at different concentrations, and neutral stimuli (i.e., no taste stimulation) by means of the Taste Strips Test. Anxiety, depression, and alexithymia were assessed. FMD patients rated the highest concentration of sweet taste (6.7 ± 2.6) as significantly more pleasant than controls (4.7 ± 2.5, *p* = 0.03), and the neutral stimuli significantly more unpleasant (patients: − 0.7 ± 0.4, controls: 0.1 ± 0.4, *p* = 0.013). Hedonic ratings were not correlated to anxiety, depression, or alexithymia scores. Hedonic response to taste is altered in FMD patients. This preliminary finding might result from abnormal interaction between sensory processing and emotional valence.

## Introduction

Functional motor disorders (FMDs) are part of the wide spectrum of functional neurological disorders that include non-epileptic seizures and sensory symptoms. FMDs are characterized by abnormal movements (e.g., weakness, tremor, and dystonia) that are clinically incongruent with motor disorders caused by known neurological diseases and significantly altered by distraction or non-physiological maneuvers including dramatic placebo response (Lehn et al. 2016). FMDs are often associated with psychiatric comorbidity, e.g., personality disorders and severe distress, disability, and social isolation (Feinstein et al. 2001; Carson et al. 2011). FMD patients have been reported to show altered sensory processing (Morgante et al. 2018), including reduced accuracy in visceral sensitivity, associated with abnormal identification of emotions (Demartini et al. 2014; Teodoro et al. 2018; Marotta et al. 2020) and changes in emotion regulation strategies (Fiess et al. 2015). Impairment of emotional processing was documented in patients with non-epileptic seizures (Nováková et al. 2015), and reduced positive emotional behavior to pleasant, neutral, and unpleasant pictures was reported in these patients (Marotta et al. 2020; Roberts et al. 2012). Indeed, alterations in neural circuits mediating emotional processing and perceptual awareness (Perez et al. 2015) and abnormal activity in the insula (Lehn et al. 2016) were reported in FMDs. Patients with FMDs often have anxiety, depression, alexithymia, and/or affect dysfunction (Pick et al. 2019). These converging pieces of evidence suggest that abnormal interaction between sensory processing and emotional valence might represent a trait of FMDs.

Given the abnormal sensory and emotional processing, and the absence of information on chemosensory processing, the present study explored responses to taste in FMDs. In particular, the study was aimed (a) to measure accuracy in recognition of different tastes, and (b) to assess the subjective hedonic experience to the presented stimuli in the patients with FMD and matched controls. To these aims, we recruited a group of FMD patients, who were assessed for gustatory perception for the four basic tastes (sweet, sour, salty, and bitter) by means of validated tests. In addition, patients were asked on the hedonic sensation (i.e., pleasant, neutral, and unpleasant) in response to each stimulus at different concentrations and neutral stimuli (i.e., no taste stimulation). Every participant underwent a psychological evaluation to explore anxiety, depression, and alexithymia.

## Methods

### Subjects

We enrolled a group of 20 patients (mean age: 44.6 ± 13.3 years, range: 16–73) with clinically definite FMD according to Gupta–Lang criteria (Gupta and Lang 2009) with disease duration of 3.4 ± 2.0 years (range: 1–7 years; Table 1). Patients were recruited and assessed at Verona University Hospital, Milan San Paolo Hospital, and Voghera Hospital, Italy. A group of 24 healthy subjects (mean age: 40.5 ± 15.7 years, range: 19–79) served as controls. The two groups were matched for age and gender. Patients and controls underwent careful history taking and clinical and neurological examination to exclude conditions that could have influenced taste evaluation. Exclusion criteria were ear nose and throat disorders (e.g., sino-nasal pathologies and middle ear surgery), recent head trauma, thyroid disorders, diabetes mellitus, asthma or allergies, gastroesophageal reflux disease, and any condition, including drugs or dietary habits, that could interfere with gustatory perception. People who were current smokers were also excluded (Ajmani et al. 2017). The study was performed in accordance with the Declaration of Helsinki and the guidelines of the local ethics committee (Comitato Etico per la Sperimentazione Clinica—CESC) and each participant gave written informed consent.

**Table 1:** Demographic and clinical features of the functional motor disorders (FMD) patients

Pt	Age (y)	Sex	Disease duration (y)	Phenomenology/body part	Other neurological functional disorders
1	44	M	1	L UL and L LL weakness	L UL and L LL somatosensory deficits
2	46	F	3	B UL and B LL weakness	Gait disturbance
3	46	F	6	B UL dystonia	–
4	47	M	1	B UL and B LL weakness	Gait disturbance
5	58	F	1	B LL and UL dystonia	–
6	48	F	3	Gait disturbance	Dysarthria
7	39	F	3	B LL weakness	–
8	47	F	3	Gait ataxia	Dysarthria
9	51	F	4	Facial disorder	–
10	36	F	2	Gait disturbance	–
11	73	F	5	Gait disturbance	–
12	67	F	5	R UL dystonia	–
13	16	F	1.5	R UL tremor	–
14	25	F	6	Gait disturbance	–
15	50	F	1	Gait disturbance	Speech disorder
16	50	F	7	R UL and R LL dystonia	–
17	42	F	3	R UL tremor and L UL and L LL weakness	–
18	25	F	2	B UL and B LL weakness	Trunk myoclonus
19	39	F	3	B UL tremor	–
20	40	F	7	B UL tremor	–

*Pt* patient’s number, *M* male, *F* female, *y* years, *R* right, *L* left, *B* bilateral, *UL* upper limb(s), *LL* lower limb(s)

### Psychological evaluation

Before the chemosensory evaluation, all patients and controls were assessed for anxiety, depression, and alexithymia by means of the following scales: Hamilton Anxiety Rating Scale (HARS), Hamilton Depression Rating Scale (HDRS), and Toronto Alexithymia Scale (TAS-20).

HARS: This scale estimates the anxiety intensity, and includes 14 items depicting anxiety-related symptoms, scored on a 5-point Likert-like scale ranging from 0 (absent) to 4 (very severe). The total score ranges from 0 to 56. A global score < 7 indicates no or minimal anxiety, a score between 8 and 14 indicates mild anxiety, while a score between 15 and 23 indicates moderate anxiety, and a score ≥ 24 points to severe anxiety (Hamilton 1959).

HDRS: This scale assesses depression by means of an interview-based rating scale and includes 21 items defining depression-related symptoms. According to the item, a clinician scored each item on a 3- or 5-point severity scale. The total score ranges from 0 to 54. A global score < 7 indicates the absence of depressive symptoms, a score between 8 and 17 states for mild depressive symptoms, a score between 18 and 24 indicates moderate severity, and a score ≥ 25 states for severe depressive symptoms (Hamilton 1960).

TAS-20: TAS-20 assesses the capacity to identify and describe feelings. It consists of 20 items bundled in three subscales: (1) difficulty identifying feelings, (2) difficulty describing feelings, and (3) externally oriented feelings. Each item receives a score on a 5-point scale ranging from 1 (strongly disagree) to 5 (strongly agree). The total score ranges from 20 to 100 and higher scores indicate more severe alexithymia (Bagby et al. 1994).

### Olfactory evaluation

Olfaction was assessed to exclude the presence of subjects with hyposmia or functional anosmia, which could have possibly interfered with taste due to the chemosensory interaction phenomena (Landis et al. 2010). Olfaction was evaluated in a well-ventilated room with the validated Sniffin’ Sticks Extended test (Burghart, Wedel, Germany) (Hummel et al. 2007) that consists of three subtests, namely odor threshold (i.e., the concentration at which the odor is reliably detected), discrimination (i.e., the ability of the subject to discriminate which odor is different among three odors, two identical and one different), and identification (i.e., the subject has to identify 16 different odors). The sum of these scores (TDI score) defines the olfactory performance *status* of subject as normosmia (TDI score ≥ 30.5), hyposmia (TDI score = 16–30.5), and functional anosmia (TDI score ≤ 16). The term “functional anosmia” indicates subjects who are completely anosmic or have some olfactory function left, which is not useful in daily life (Kobal et al. 2000). A subject with functional anosmia may still have a residual olfactory function, but this remnant perception does not contribute to the enjoyment during meals or to detection of, e.g., rotten food or gas leaks. Both for threshold and discrimination tests, subjects were tested blindfolded with a sleeping mask to prevent the visual identification of the odorant-containing pens. During the identification test, the subject has to identify 16 common odors (e.g., orange, peppermint, coffee, fish, and banana), choosing the correct answer among a list of four options each time. To increase the reliability of the measurements, for every subtest, each subject was asked to give an answer with a forced-choice paradigm (Hummel et al. 2007).

### Gustatory evaluation and gustatory main task

Taste was first examined by the Whole Mouth Test (WMT) and then with the Taste Strips Test (TST, Burghart Company, Wedel, Germany) (Mueller et al. 2003; Landis et al. 2009). The WMT consists of four supra-threshold taste solutions (sweet, sour, salty, and bitter) sprayed into the oral cavity for having a first indication on gustatory function and to rule out gross taste dysfunction. TST is a validated examination procedure for the determination of gustatory sensitivity with four concentrations. We used 16 spoon-shaped filter papers impregnated with four concentrations (labeled 1, 2, 3, 4) of the four basic taste solutions (sweet: 0.05, 0.1, 0.2, 0.4 g/ml sucrose; sour: 0.05, 0.09, 0.165, 0.3 g/ml citric acid; salty: 0.016, 0.04, 0.1, 0.25 g/ml sodium chloride; bitter: 0.0004, 0.0009, 0.0024, 0.006 g/ml quinine hydrochloride). The strips were placed on the tongue and the subject had to identify the taste quality from a list of four descriptors (sweet, sour, salty, and bitter) in a forced-choice manner. The sum of correct identifications defines the taste performance *status* as normogeusia (TST score ≥ 9), hypogeusia (TST score < 9), and ageusia (no sensation to the highest concentrations of all the four taste solutions). Umami taste (the fifth taste) was not included in the assessment, because it is still little familiar in European countries (Landis et al. 2009; Cecchini et al. 2019). Two additional strips of the test were not impregnated with taste solutions (neutral stimuli) and could be randomly presented.

Main task: for every TST strip, including neutral ones, participants were asked to give a hedonic rating on a scale ranging from − 10 to + 10, where − 10 corresponded to “very unpleasant”, + 10 to “very pleasant”, and 0 to “neither pleasant nor unpleasant”.

### Statistical analysis

All tests were carried out with the IBM SPSS version 20.0 statistical package. The normality of distribution was analyzed with the skewness-kurtosis test. Continuous variables were explored with *t* test in case of normal distribution, and non-parametrical Mann–Whitney *U* test when the distribution was not normal. Pearson’s chi-square test with Yates’ correction was applied to dichotomous variables. For hedonic scores, two-way repeated-measure ANOVA was used with the between-subject factor GROUP (patients, controls) and the within-subject factor CONCENTRATION (coded as 1, 2, 3, 4) for each type of stimulus (sweet, sour, salty, and bitter). Post hoc comparisons were done by means of *t* test for paired and independent samples. A three-way ANOVA model including also the within-subject factor TASTE (sweet, sour, salty, and bitter) was not feasible because of the high number of wrong identification responses. Correlation between hedonic scores to taste stimuli and psychological variables was evaluated with the Spearman’s *ρ* correlation test. For all the tests, *p* < 0.05 (two-tailed, with Bonferroni’s correction for post hoc comparisons if needed) was taken as the significance threshold for all the tests.

## Results

### Psychological evaluation

HARS, HDRS, and TAS scores were within normal range in all controls.

In FMD patients, the mean HARS was 9.1 ± 6.9 (range: 0–25), indicating on average mild anxiety, with only one patient with severe anxiety and two patients reporting moderate anxiety. The mean HDRS was 10.7 ± 6.8 (range: 0–22), indicating on average mild depression, with five patients reporting moderate depression. The mean TAS was 48.9 ± 12.2 (range: 30–73).

### Olfactory and gustatory evaluation

All patients and controls were normosmic and normogeusic, as TDI and TST scores were in the normal range in all subjects. The preliminary evaluation of gross gustatory function by means of the WMT spray solutions was normal in all participants. All subjects correctly identified the four supra-threshold taste solutions. The mean TDI and TST scores did not differ between the two groups (Table 2).

**Table 2:** Demographic characteristics and olfactory scores of participants

	Patients (*N* = 20)	Controls (*N* = 24)	*p* value
Age	44.6 ± 13.3	40.5 ± 15.7	n.s.
Sex (M/F)	2/18	7/17	n.s.
TDI score	34.7 ± 3.8	35.3 ± 3.6	n.s.
TST score	12.5 ± 1.9	12.7 ± 2.0	n.s.

Data are presented as mean ± S.D

*TDI* total score to the Sniffin’ Sticks Extended test (normal values: ≥ 30.5), *TST* Taste Strip Test total score (normal values: ≥ 9), *n.s.* not significant

### Hedonic score to taste stimuli

The number of correct and wrong identifications to each type of taste stimuli was not significantly different between the two groups (Pearson’s chi-square test: *p* > 0.05 for all comparisons), being the number of correctly identified responses larger to higher concentrations (Table 3). Only the hedonic scores for correctly identified stimuli were considered for further analyses.

**Table 3:** Number of correct identifications to taste stimuli at different concentrations

	Sweet stimuli (sucrose concentration)			
	0.05 g/ml	0.1 g/ml	0.2 g/ml	0.4 g/ml
Patients (*N* = 20)	10 (50%)	19 (95%)	19 (95%)	18 (90%)
Controls (*N* = 24)	16 (67%)	21 (88%)	24 (100%)	24 (100%)

	Sour stimuli (citric acid concentration)			
	0.05 g/ml	0.09 g/ml	0.165 g/ml	0.3 g/ml
Patients (*N* = 20)	0 (0%)	10 (50%)	16 (80%)	17 (85%)
Controls (*N* = 24)	3 (13%)	14 (58%)	18 (75%)	24 (100%)

	Salty stimuli (sodium chloride concentration)			
	0.016 g/ml	0.04 g/ml	0.1 g/ml	0.25 g/ml
Patients (*N* = 20)	14 (70%)	17 (85%)	16 (80%)	18 (90%)
Controls (*N* = 24)	13 (54%)	19 (79%)	19 (79%)	22/2

	Bitter stimuli (quinine hydrochloride concentration)			
	0.0004 g/ml	0.0009 g/ml	0.0024 g/ml	0.006 g/ml
Patients (*N* = 20)	15 (75%)	19 (95%)	18 (90%)	19 (95%)
Controls (*N* = 24)	17 (71%)	22 (92%)	22 (92%)	23 (96%)

Data are presented as percentage (%) of correct responses for identification.

**Fig. 1:**
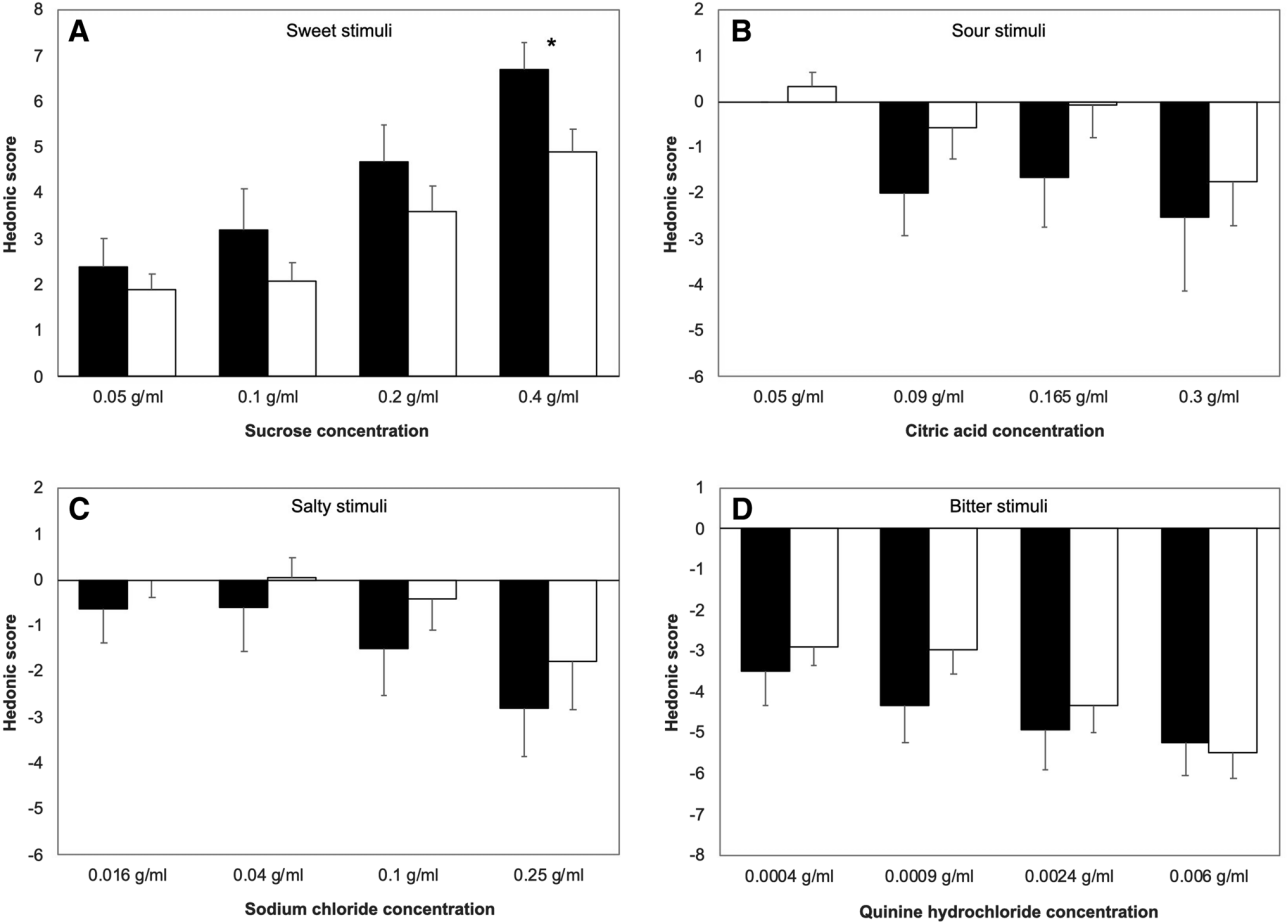
Hedonic score to sweet (panel **a**), sour (panel **b**), salty (panel **c**), and bitter (panel **d**) taste stimuli in patients (closed boxes) and controls (open boxes). *Marks *p* < 0.05. Vertical error bars equal 1 SEM

Overall, FMD patients scored the hedonic valence of tastes more pleasant/unpleasant than controls (Fig. 1).

For sweet stimuli, ANOVA showed a significant effect of CONCENTRATION (*F* = 14.5, *p* < 0.001) and GROUP (*F* = 5.81, *p* = 0.021) being the rating significantly more pleasant in patients than in controls for the highest sucrose concentration (0.4 g/ml sucrose; patients: 6.7 ± 2.6, controls: 4.7 ± 2.5, Mann–Whitney *U* test: *p* = 0.03; Fig. 1), but no significant interaction. For bitter and salty stimuli, ANOVA showed a significant effect of CONCENTRATION (bitter: *F* = 6.3, *p* = 0.003; salty: *F* = 5.5, *p* = 0.033), but no effect of GROUP or significant interaction (Fig. 1). For sour stimuli, ANOVA yielded neither significant factors nor interactions.

**Fig. 2:**
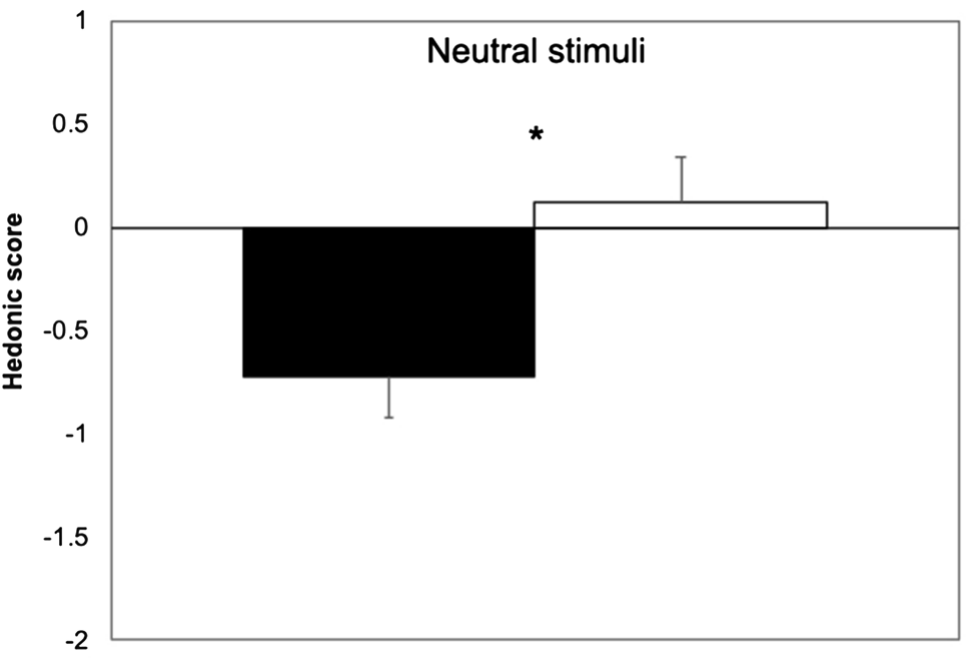
Hedonic score to neutral taste stimuli in patients (closed boxes) and controls (open boxes). *Marks *p* < 0.05. Vertical error bars equal 1 SEM

Hedonic score to neutral stimuli differed significantly between patients and controls (Mann–Whitney *U* test: *p* = 0.013; Fig. 2), being the rating significantly more unpleasant in the first group than in the latter.

More extreme ratings from both pleasant, unpleasant categories, and neutral stimuli were not provided by the same subjects.

Hedonic scores to the highest concentrations of the four basic taste solutions and to neutral stimuli were not correlated to HARS, HDRS, or TAS scores. For the other concentrations, no correlation analysis was performed because of the high number of wrong responses.

## Discussion

This work explored the hedonic response to the administration of the four basic tastes at different concentrations and to neutral taste stimuli in a group of FMD patients compared to healthy subjects. The main findings of the study were that patients rated: (a) sweet stimuli as significantly more pleasant to the highest sucrose concentration; (b) neutral stimuli as significantly more unpleasant than controls.

The oral tissues have a strong somatosensory innervation, and they are the site of some of our most intense and vivid bodily experiences. The strong link between taste and its hedonic value is well known (Steiner et al. 2001). Emotional states of pleasantness or unpleasantness to taste contribute to guide our food selection and protect us from potentially harmful foods, modulating our behavior. Sugars determine the pleasant sensory quality of sweetness, as sweet taste is generally associated with energetic food. In contrast, unpleasant valence associated with bitter sensation is associated with many molecules and substances that are potentially poisonous or toxic (Chandrashekar et al. 2006; Beauchamp 2016).

The first finding of the study was that, when presenting stimuli of different concentrations to patients, we documented a more pleasant response to sweet stimuli that were significant for higher sucrose concentration in patients compared to controls, but no significant difference to any concentration for the other taste stimuli. These findings suggest that FMD patients may overstate the valence of positive taste stimuli. To the best of our knowledge, this is the first study on chemoreception in FMDs and these findings are difficult to compare to those from studies with the other types of sensory modalities, because of the specific features of taste sensory systems in the brain. These findings, even preliminary, appear to be in keeping with and extend those from a previous study that showed the avoidance of negative stimuli, specifically sad faces, in FMD patients (Marotta et al. 2020). Our data suggest an overstatement of the valence of positive stimuli. However, our results on sweet stimuli should be considered with caution, as it could also be explained by the relatively larger number of hedonic ratings to sweet stimuli compared to the relatively smaller number of correctly identified ratings to non-sweet stimuli.

The second finding of our study was that neutral stimuli were scored with a significantly more negative valence by patients than controls. When the filter paper strip without a gustatory substance inside is administered into the oral cavity, somatic afferents are triggered. The oral cavity is among the most richly innervated areas of the human body, in terms of the number and variety of receptors (i.e., mechanoreceptors, nociceptors, and thermoreceptors) and here a wide range of somatosensory signals originate (Haggard and Boer 2014). At this level, the integration and combination of these afferents are important, so that the oral somatosensory awareness represents a complex issue (Haggard and Boer 2014). The oral cavity is considered as a particularly developed and specialized sensory system (Linden 1990; Jacobs et al. 2002; Zhou et al. 2018) where taste and somatosensory afferents are closely linked, so that some gustatory stimuli can also induce tactile and thermal sensation (Green and Hayes 2003).

We may hypothesize that neutral strips could have activated somatosensory afferents in FMD patients, thus leading to a negative valence, in keeping with the presence of somatosensory functional symptoms in the majority of them.

However, perception and valence of taste are also related to other elements linked to the somatosensory domain, such as olfaction, vision, satiety/hunger (Rolls 2000; Chandrashekar et al. 2006), as well as factors related to the subject (e.g., experience, preference, and internal state) (Hummel and Welge-Lüssen 2006), and to the context where the stimulus is presented. All these aforementioned features could not be assessed in our experimental setting.

Our data are in keeping with the previous literature, which reported sensory and emotional processing to be altered in FMD patients, so that this impaired sensory–emotional substrate might be a factor leading to the abnormal hedonic rating we found. They are in accordance to the Feldmann Barrett’s emotion theory, whereby emotions are cognitive processes linked to interoception and are explained using the predictive and attentional mechanisms (Hoemann and Feldman 2019), i.e., the same principles that apply for functional symptoms development according to some of the recent neurobiological models of FMD (Keynejad et al. 2020). We may hypothesize that an abnormal emotion evaluation calibration, as observed in this study, may result from altered perception, i.e., construction of taste representations and subjective experience of hedonic pleasure.

Our results are also in accordance with functional neuroimaging studies that showed increased activity of limbic and paralimbic regions (Aybek et al. 2015; Espay et al. 2018; Demartini et al. 2019), and alterations in the amygdala, orbitofrontal cortex, and the insula, associated with the abnormal emotional processing in FMD (Voon et al. 2010; Pick et al. 2019). These regions contribute to the gustatory central process, in terms of quality of the perception, intensity, reward value, and hedonicity rating. Moreover, the previous animal studies showed that sweet and bitter cortical projections to the amygdala segregate in different regions, where distinct populations of neurons exist, so that sweet and bitter central process could be considered anatomically different (Cai et al. 2014; Namburi et al. 2015; Gore et al. 2015; Kim et al. 2016, 2017; Douglass et al. 2017; Han et al. 2017; Beyeler et al. 2018; Wang et al. 2018). Furthermore, a very recent work showed that the firing rate of neurons in the insula is modulated by stimulus concentration instead of stimulus type (Porcu et al. 2020). These pieces of information might explain the specificity of the abnormal valence score to sweet but not bitter stimuli, and to higher concentrations only, in FMD patients.

Indeed, the dissociation between sensory–discriminative (i.e., preserved stimulus recognition) vs. cognitive–emotional (i.e., altered subjective hedonic evaluation of the stimuli) in our FMD patients is in keeping with a previous study, where no differences were found in tactile and pain thresholds, but pain tolerance was increased in patients with functional dystonia (Morgante et al. 2018).

We found no correlation between hedonic scores and anxiety, depression, and alexithymia, thus arguing against these factors as variables that could contribute to our results.

Despite the aforementioned links with the previous anatomical and imaging studies, it is important to mention that our work is preliminary and further investigations are needed. The study has a number of limitations, including the small sample size with many wrong identification responses, the unbalance condition (i.e., two trials) for neutral stimuli, the variety of symptoms across FMD patients, the absence of information on oral somatosensory function, the absence of neuroimaging data and other biomarkers, and the presence of few neuropsychological data in the absence of correlation. Further studies should confirm these preliminary findings in a larger sample of FMD patients. Additional functional neuroimaging and neurophysiological data could strengthen the hypothesis that hyperactivation of some gustatory amygdala and insular regions might underscore the higher hedonic value to higher sucrose stimuli in FMD patients (Pick et al. 2019).

## Data Availability

A full dataset of data and statistical code is available from corresponding authors at reasonable request contingent on approval from the Ethical Committee (Comitato Etico per la Sperimentazione Clinica—CESC).
